# Implementation of Blood‐based Biomarkers in a Large Dementia Care Clinic: Case‐based Report from the UCSF Memory and Aging Center

**DOI:** 10.1002/alz.090790

**Published:** 2025-01-09

**Authors:** Andrew Breithaupt, Nhat Minh Bui, Yasmeen Gonzalez, Renaud La Joie, Peter A. Ljubenkov, Katherine L Possin, Julio C. Rojas, David N. soleimani‐meigooni, Adam M. Staffaroni, Melanie Stephens, Elena Tsoy, Charles Windon, Adam L. Boxer, Gil D. Rabinovici, Bruce L. Miller, Lawren VandeVrede, Tara Ellingson

**Affiliations:** ^1^ Memory and Aging Center, Weill Institute for Neurosciences, University of California, San Francisco, San Francisco, CA USA; ^2^ Memory and Aging Center, Weill Institute for Neurosciences, University of California, San Francisco (UCSF), San Francisco, CA USA; ^3^ Memory & Aging Center, Department of Neurology, University of California in San Francisco, San Francisco, CA USA; ^4^ Memory and Aging Center, Weill Institute for Neurosciences, University of California, San Francisco, CA USA

## Abstract

Several new blood‐based biomarkers (BBMs) of Alzheimer’s disease and related neuropathologies have completed late‐stage validation and are beginning to be used in the clinical setting. However, the role of BBMs in various clinical settings, especially their specific context‐of‐use, remains an open question for the field. Several studies are beginning to systematically collect information on BBM real‐word diagnostic performance, but as BBMs transition from research settings to clinical implementation, experience from clinical providers in specialty centers can inform and guide use. In this session, BBM implementation at a large specialty care center, the University of California San Francisco (UCSF) Memory and Aging Center (MAC), will be discussed. The UCSF MAC is a division of the Department of Neurology dedicated to the diagnosis and care of patients with cognitive problems and their families, and the MAC has 32 clinicians who collectively evaluate over one thousand new patients a year with cognitive concerns. Additionally, the MAC recently launched an Alzheimer’s Infusion Core, dedicated to evaluating patients for the newly approved disease‐modifying treatments (DMT). Within the context of the UCSF MAC, integration of BBMs into clinical care will be reviewed using a case‐based format, including a description of overall provider usage, impact on differential diagnosis and subsequent management, and perceived role in the work‐up for patients interested in DMTs. Additional discussion will touch upon logistics and challenges to implementation, with a road map for future clinical integration.

